# Trends in SAVR with biological vs. mechanical valves in middle-aged patients: results from a French large multi-centric survey

**DOI:** 10.3389/fcvm.2023.1205770

**Published:** 2023-08-28

**Authors:** Thierry Caus, Yuthiline Chabry, Joseph Nader, Jean François Fusellier, Jean Louis De Brux, for the EpiCard investigators, Paul Achouh

**Affiliations:** ^1^ Department of Cardiac Surgery University Hospital Amiens-Picardie, Amiens, France; ^2^ Laboratoire MP3CV-University Picarde Jules Vernes-UR7517, Amiens, France; ^3^ Department of Cardiac Surgery University Paris Diderot, Paris, France; ^4^ Department of Thoracic and Cardiovascular Surgery, Clinique du Millénaire, Montpellier, France; ^5^ Department of Cardiovascular Surgery, University Hospital Pontchaillou, Rennes, France; ^6^ Department of Cardiac Surgery, University Hospital of Angers, Angers, France

**Keywords:** aortic valve, surgical aortic replacement, mechanical and biological prosthetic valves, trend, database, France, bioprosthesis, mechanical prosthesis

## Abstract

**Background/introduction:**

Currently, despite continued issues with durability ( 1), biological prosthetic valves are increasingly chosen over mechanical valves for surgical aortic valve replacement (SAVR) in adult patients of all ages, at least in Western countries. For younger patients, this choice means assuming the risks associated with a redo SAVR or valve-in-valve procedure.

**Purpose:**

To assess the use of mechanical vs. biological valve prostheses for SAVR relative to patient's age and implant time in a large population extracted from the French National Database EPICARD.

**Methods:**

Patients in EPICARD undergoing SAVR from 2007 to 2022 were included from 22 participating public or private centers chosen to represent a balanced representation of centre sizes and geographical discrepancies. Patients with associated pathology of the aorta (aneurysm or dissection) and requiring a vascular aortic prosthesis were excluded. Comparisons were made amongst centers, valve choice, implant date range, and patient age.

**Results:**

We considered 101,070 valvular heart disease patients and included 72,375 SAVR (mean age 71.4 ± 12.2 years). We observed a mechanical vs. biological prosthesis ratio (MBPR) of 0.14 for the overall population. Before 50 years old (y-o), MBPR was >1.3 (*p* < 0.001) while patients above 60 years-old received principally biological SAVR (*p* < 0.0001). Concerning patients between 50 and 60 years-old patients, MPVR was 1.04 (*p* = 0.03). Patients 50–60 years-old from the first and second study duration quartile (before August 2015) received preferentially mechanical SAVR (*p* < 0.001). We observed a shift towards more biological SAVR (*p* < 0.001) for patients from the third and fourth quartile to reach a MBPR at 0.43 during the last years of the series. Incidentally, simultaneous mitral valve replacement were more common in case of mechanical SAVR (*p* < 0.0001), while associated CABGs were more frequent in case of biological SAVR (*p* < 0.0001).

**Conclusion:**

In a large contemporary French patient population, real world practice showed a recent shift towards a lower age-threshold for biological SAVR as compared to what would suggest contemporary guidelines.

## Introduction

Currently, despite continued issues with durability (1), biological prosthetic valves are increasingly chosen over mechanical valves for surgical aortic valve replacement (SAVR) in adult patients of all ages, at least in Western countries. For younger patients, this choice means assuming the risks associated with a redo SAVR or valve-in-valve procedure.

Mechanical prosthetic valves (MPVs), for which oral direct Factor Xa inhibitors failed to be a valid option (2, 3), are not generally considered in patients above 65 years-old, even if a long-standing anticoagulant therapy is required and despite the fact that acceptable outcomes with a MPV may be achieved in selected elderly patients (4). To avoid imposing to a young patient a life-long anticoagulant therapy whenever a Ross procedure or an aortic valvular repair is out of reach, a BPV in aortic position is often perceived to be the best second choice. Thus, in Western countries and despite consensus recommendations (5–7), BPVs are nowadays implanted in a significant proportion of young and middle-aged patients requiring SAVR (8) therefore assuming the risks associated with a subsequent redo SAVR or with a valve-in-valve trans catheter aortic valve replacement (ViV-TAVR). Concerning TAVR, lowering the age-threshold and extending indications towards low-risk patients will further amplify this global move towards BPV with an exponential rise (9).

Potentially as a consequence of results of TAVR in patients at intermediate risk (10), we aimed at illustrate how much, in France, the pendulum has reached towards BPVs especially for middle-aged patients requiring a SAVR during the past 15 years. We therefore launched a large multi-centric survey to assess the use of mechanical vs. biological valve prostheses for SAVR relative to patient's age and implant time in a large population extracted from the French National Database EPICARD.

## Material and methods

Starting 20 years ago, the French National Database EPICARD is hosted on a central computer server headed by the Federation of Medical Specialities, Issy les Moulineaux, France and is prospectively implemented via internet by each logged-in participant through a dedicated e-Form including pre, per and post operative data. There has been no restriction, in France, to the availability of devices and funding for world wide marketed devices or for warfarin and other anticoagulant therapies during the study's period of time and physicians had full influence on which devices they used.

### Ethics

The EPICARD database was declared to the French National Commission for Data Protection and Liberties (CNIL ⋕1221925). Patients were informed of the potential utilization of their anonymized data for scientific purpose and could ask that their case would not be entered into the dataset. Provided that no commercial use of the data would be made and that no indication of trade mark or label of any specific prosthetic valves used would be published, our survey was approved by the Scientific Committee of the French National Database EPICARD. The study was performed in accordance with the ethical standards of in the 1964 Declaration of Helsinki and its later amendments. Use of all data strictly followed the General Data Protection Regulations.

### Study population

**Figure 1:**
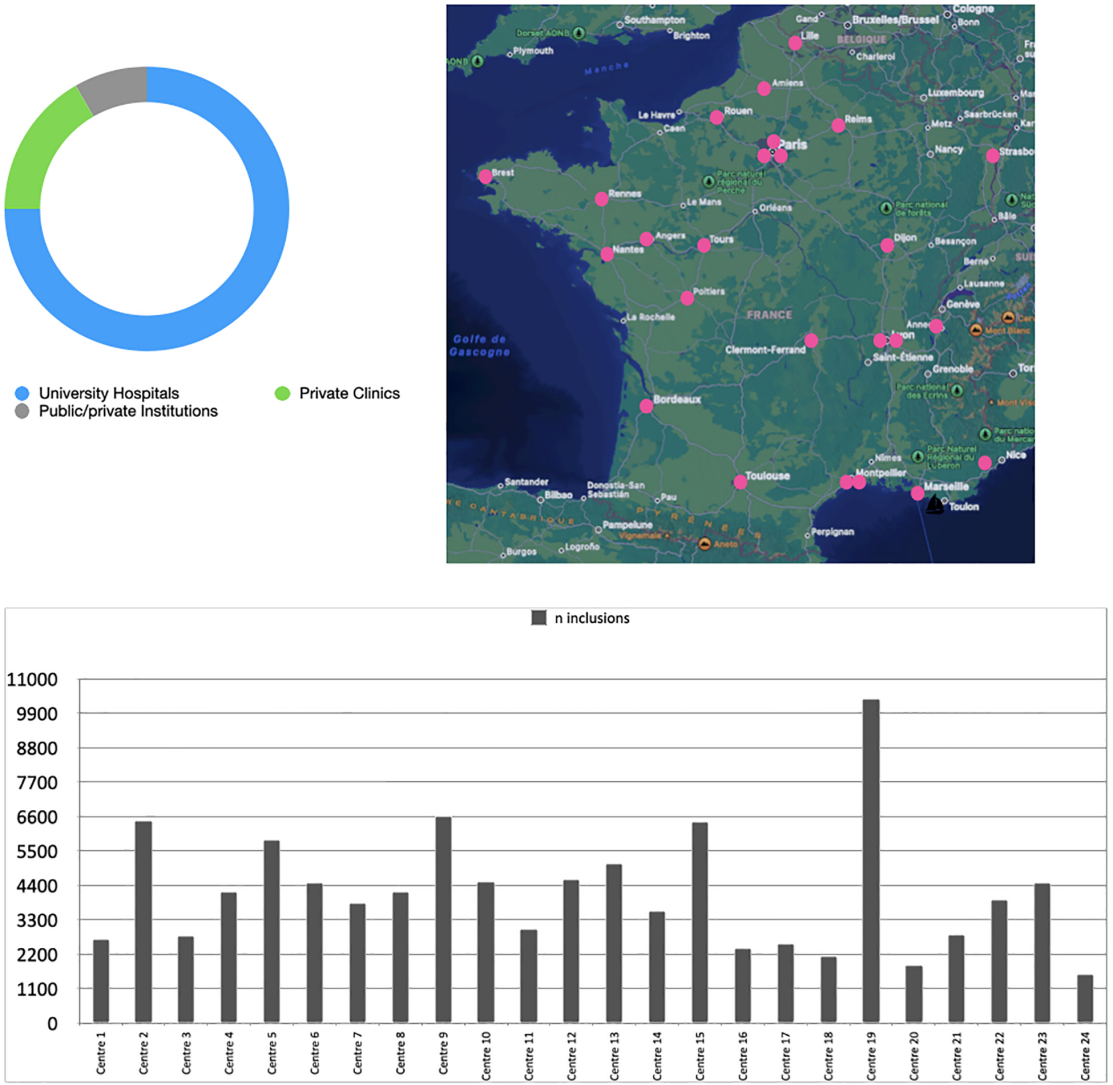
Carbon-Free survey, distribution of participating centres.

Patients in EPICARD undergoing SAVR from 2007 to 2022 were included from 22 participating public or private centers chosen to represent a balanced representation of centre sizes and geographical discrepancies (Figure 1). Patients aged under 19 years-old or patients with associated pathology of the aorta (aneurysm or dissection) and requiring a vascular aortic prosthesis were excluded. Comparisons were made amongst centers, valve choice, implant date range, and patient age.

Data on preoperative (demographic, clinical and echocardiographic) characteristics, perioperative characteristics, EuroSCORE, and in-hospital mortality/morbidity after cardiac surgery were collected for each patient. Patients who underwent an associated valvular procedure or associated CABGs were included. Anonymized data were extracted under a Excel-sheet format which allowed for further statistical analysis with MedCalc. Means are expressed with SD deviation, rates are expressed with their Poisson 95% Confidence Interval (95% CI). Continuous and categorial variables were compared with a *t*-test, a Wilcoxon rank-sum test, or a Pearson *χ*^2^-statistic whenever required. Tests were based on a two tailed *p*-value for an α-risk at 5%. Because data about long term follow-up remain seldom in the EPICARD database, results were restricted to the in-hospital period. Patients were categorized into duration quartiles according to the date of operation: Q1 (18,093 patients) between 01/01/2007 and 24/10/2011; Q2 (18,093 patients) between 24/10/2011 and 02/07/2015; Q3 (18,093 patients) between 02/07/2015 and 29/01/2019; Q4 (18,096 patients) between 29/01/2019 and 31/12/2022.

## Results

### Population

**Figure 2:**
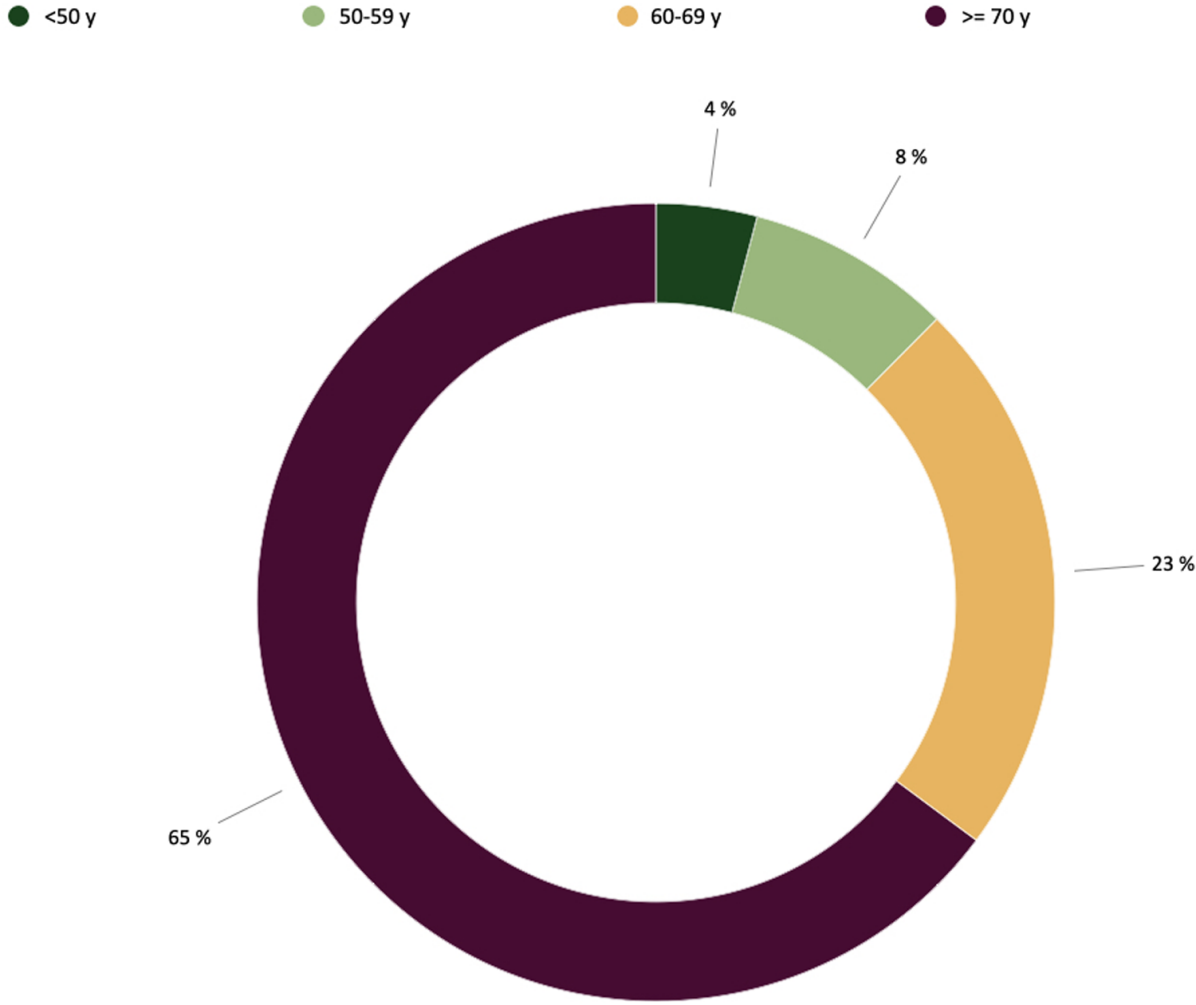
Proportion of patients with regards to age for each type of valvular prothesis in aortic position according to the ESC and AHA-ACC recommendations.

Out of 411,375 patients entered into the EPICARD Database during the study period, a population still including a very large amount of patients operated on for CABG only, we considered 101,070 valvular heart disease patients and included 72,375 SAVR (mean age 71.4 ± 12.2 years) 26,185 of which were women (36.1%). SAVR was performed as a redo surgery in 5,434 cases (7.5%). SAVR was an isolated procedure in most cases and was associated with another valve procedure in 7,123 cases (9.8%) or with CABG in 15,621 cases (21.6%). A total of 63,466 patients (87.7%) received a BPV and 8,909 patients received a MPV. Therefore we observed a mechanical vs. biological prosthesis ratio (MBPR) of 0.14 for the overall population. Patient's preoperative characteristics for each prosthetic valve type are detailed in Table 1 and overall age distribution where percentages are representative of the proportion of patients from our population in each class of age is presented in Figure 2.

**Table 1:** Preoperative characteristics at admission and echocardiography.

	Mechanical valves 8,909	Biological valves 63,466	*p*
Age, mean. (SD)	57.1 (11.6)	73.5 (10.9)	<0.0001
Women, No. (%; 95% CI)	2,841 (31.9; 30.7–33.1)	23,344 (36.8; 36.3–37.3)	<0.0001
BMI (kg/m^2^), mean. (SD)	27.91 (10.73)	27.79 (8.76)	0.2
EuroSCORE II, mean. (SD)	2.83 (5.2)	3.96 (6.1)	<0.0001
Left ventricular ejection fraction (LVEF), mean. (SD) LVEF < 30 (%; 95% CI)	58.4 (12.4)	58.6 (11.9)	0.31
	276 (3.1)	1,901 (3)	0.72
Systolic pulmonary arterial pressure (SPAP), mmHg, mean (SD) SPAP > 55 mmHg, No. (%; 95% CI)	32.9 (11.9)	33.8 (12.9)	<0.0001
	490 (5.5; 5–6)	3,053 (4.8; 4.6–4.9)	0.006
Previous AF (%; 95% CI)	62 (0.7; 0.5–0.8)	1,495 (2.36; 2.23–2.47)	<0.0001
Previous Anticoagulation (%; 95% CI) VKA	645 (7.2; 6.6–7.8)	4,800 (7.6; 7.3–7.8)	0.27
Direct Anticoagulant	38 (0.4; 0.3–0.6)	1,091 (1.7; 1.6–1.8)	<0.0001
Diabetes (%; 95% CI)	1,483 (16.6; 15.8–17.5)	14,055 (22.1; 21.8–22.5)	<0.0001
Extracardiac arteriopathy (%; 95% CI)	711 (7.9; 7.4–8.5)	7,765 (12.2; 12–12.5)	<0.0001
COPD (%; 95% CI)	554 (6.2; 5.7–6.7)	4,953 (7.8; 7.6–8)	<0.0001
Endocarditis (%; 95% CI)	506 (5.7; 5.2–6.1)	2,626 (4.1; 3.9–4.3)	<0.0001
Aortic regurgitation None, No. (%; 95% CI)	4,238 (47.5; 46.1–49)	40,113 (63; 62.6–63.8)	<0.0001
Mild, No. (%; 95% CI)	932 (10.5; 9.8–11.1)	6,477 (10.2; 9.9–10.5)	0.47
Moderate, No. (%; 95% CI)	1,131 (12.7; 11.9–13.4)	4,366 (6.9; 6.7–7.1)	<0.0001
Severe, No. (%; 95% CI)	1,460 (16.4; 15.6–17.2)	5,068 (8.4; 7.7–9)	<0.0001
Mitral regurgitation None, No. (%; 95% CI)	7,823 (87.8; 85.9–89.8)	58,134 (91.6; 90.9–92.3)	0.0005
Mild, No. (%; 95% CI)	179 (2; 1.7–2.3)	1,506 (2.3; 2.2–2.5)	0.03
Moderate, No. (%; 95% CI)	385 (4.3; 3.9–4.7)	1,442 (2.2; 2.1–2.4)	<0.0001
Severe, No. (%; 95% CI)	387 (4.3; 3.9–4.8)	1,420 (2.2; 1.8–2.4)	<0.0001
Tricuspid regurgitation None, No. (%; 95% CI)	8,504 (95,4; 93.4–97.5)	61,458 (96.8; 96.1–97.6)	0.21
Mild, No. (%; 95% CI)	73 (0.8; 0.6–1)	443 (0.7; 0.6–0.8)	0.2
Moderate, No. (%; 95% CI)	134 (1.5; 1.3–1.7)	513 (0.8; 0.7–0.9)	<0.0001
Severe, No. (%; 95% CI)	147 (16.5; 14–19.4)	399 (6.3; 5.6–6.9)	<0.0001
Aortic stenosis (%; 95% CI)	5,856 (65.7; 64–67.4)	49,525 (78; 77–79)	<0.0001
Mitral stenosis (%; 95% CI)	609 (6.8; 6.3–7.4)	1,309 (2; 1.9–2.1)	<0.0001

#### Proportion of biological vs. mechanical prosthesis according to age

Out of the 8,909 patients who received a mechanical prosthetic valve, most patients who received a mechanical SAVR (6,913) were under 50 years-old. In this class of age, MBPR was >1.3 (*p* < 0.001) while patients above 60 years-old received principally biological SAVR (*p* < 0.0001). Concerning patients between 50 and 60 years-old patients, MPVR was 1.04 (*p* = 0.03). Patients 50–60 years-old from the first and second study duration quartile (before August 2015) received preferentially mechanical SAVR (*p* < 0.001). We observed a shift towards more biological SAVR (*p* < 0.001) for patients from the third and fourth quartile to reach a MBPR at 0.43 during the last years of the series.

**Figure 3:**
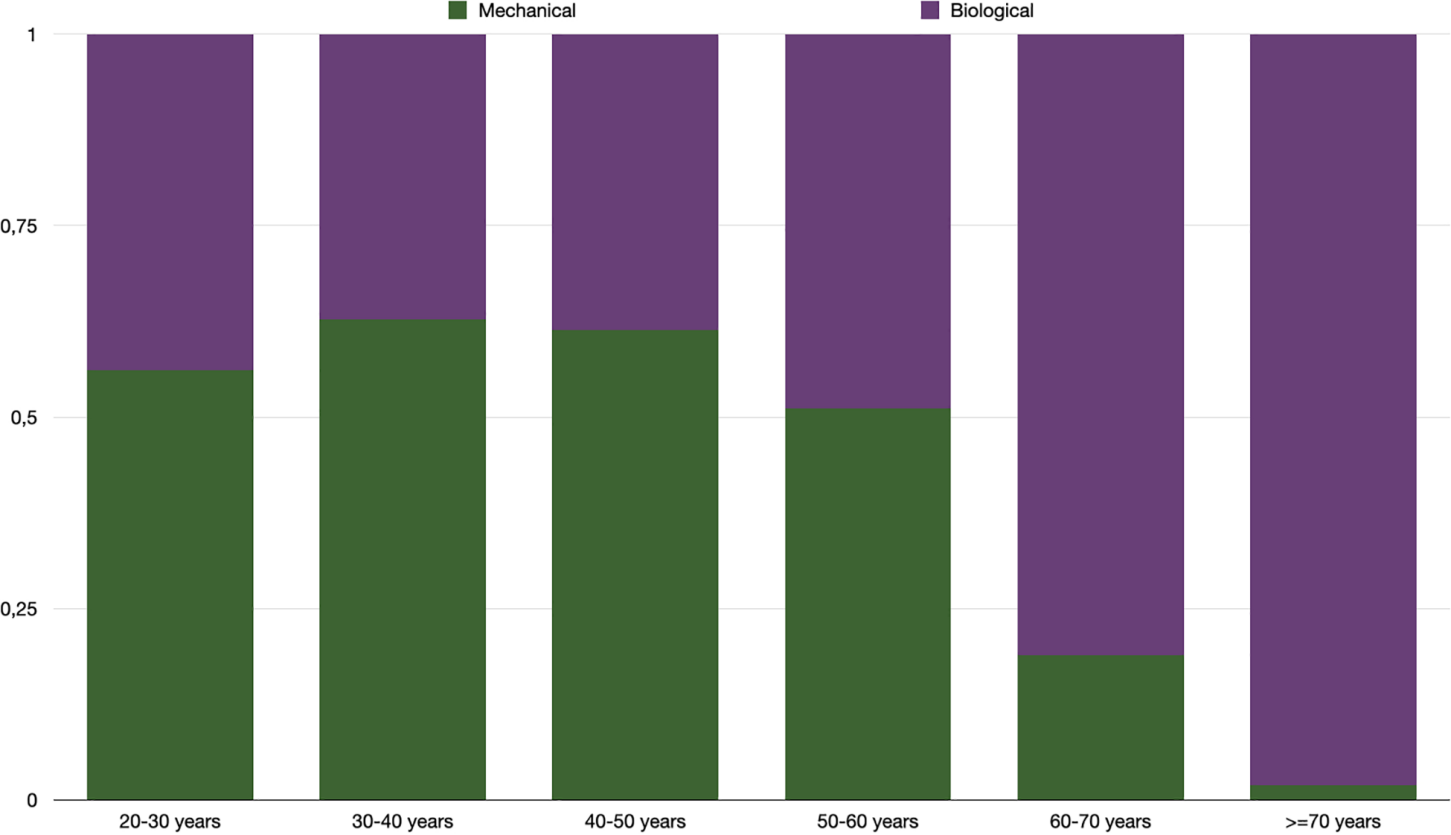
Proportion of SAVR with mechanical versus biological prosthetic valve per class of age during the overall study time (2007–2022).

**Figure 4:**
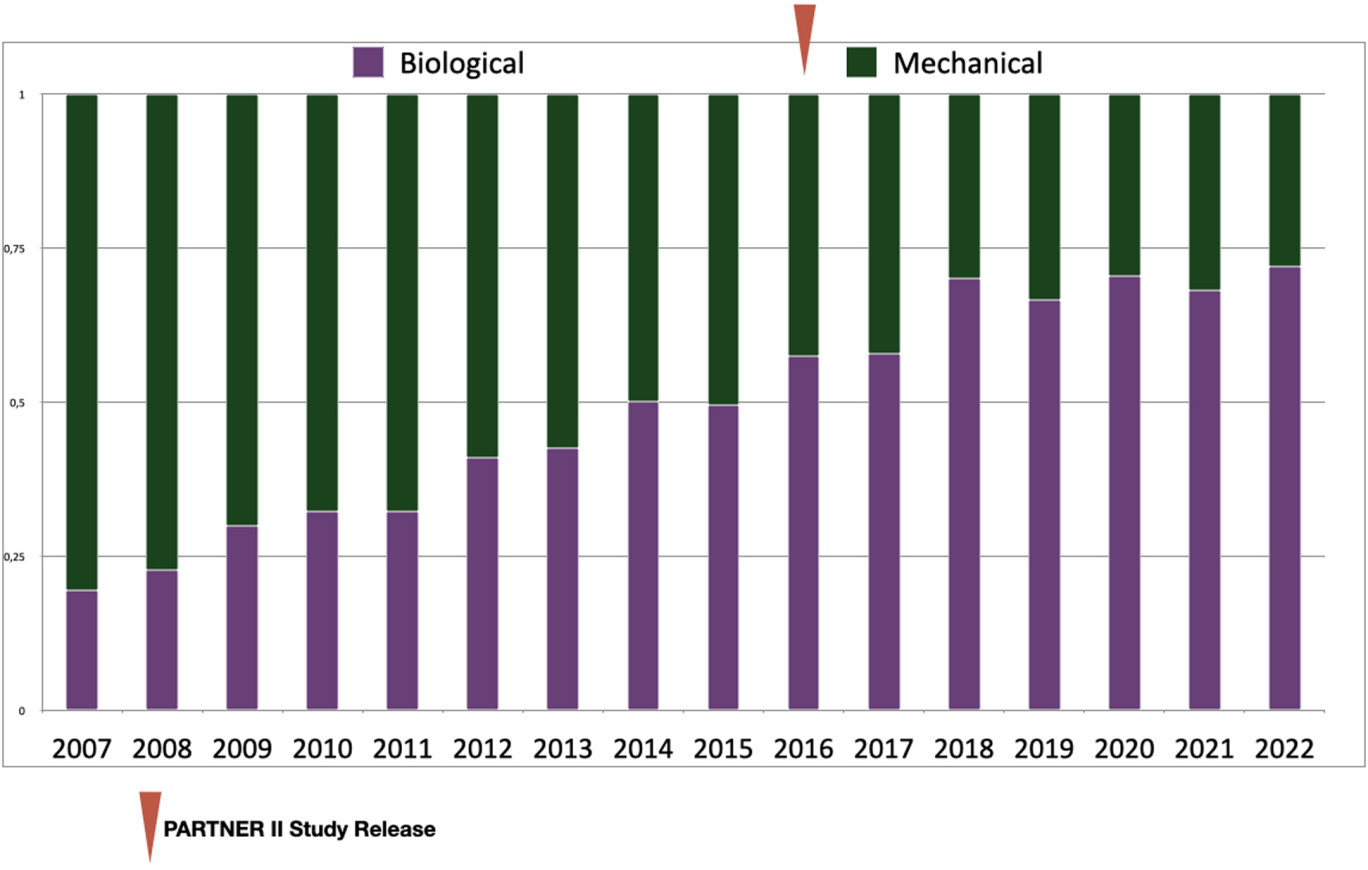
Proportion of SAVR with biological versus mechanical prosthetic valves for patients between 50 and 60 years-old per year during study time (2007–2022).

Figure 3 describes the distribution for each type of valvular prothesis per age decade throughout the overall 15 years of the survey. Figure 4 shows the trend across time of mechanical vs. biological SAVR in this sub-group population together with a landmark of the Partner II study (10) which may have affected the implantation policy.

#### Proportion of biological vs. mechanical prosthesis according to other factors

**Figure 5:**
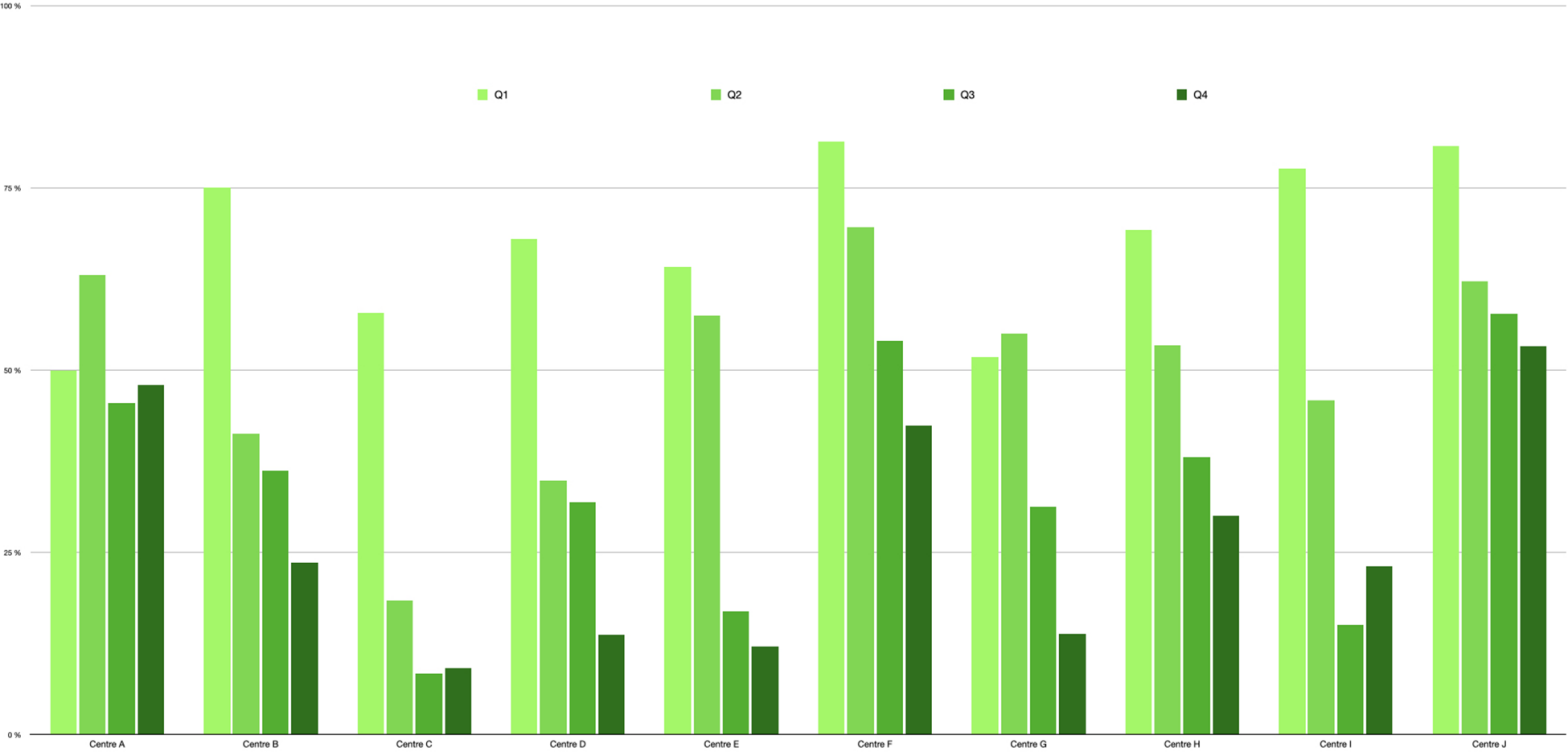
Proportion of mechanical valve for patients operated on for SAVR between 50 and 60 years-old per centre (sample) and per quartile during study time (2007–2022). Patients operated on between 01/01/2007 and 24/10/2011 (Q1); between 24/10/2011 and 02/07/2015 (Q2); between 02/07/2015 and 29/01/2019 (Q3) and between 29/01/2019 and 31/12/2022 (Q4).

A significantly higher proportion of women received BPVs. Though childbearing considerations might have play a role, sex was however not a criteria of choice in patients under 40 years old for which 63% women vs. 60% men received a MPV in aortic position (NS). Probably more a consequence of an older age than a criteria of choice, patients receiving a BPV had more co-morbidity (Table 1) like diabetes, peripheral arteriopathy or chronic obstructive pulmonary disease (*p* < 0.0001) and a higher EuroSCORE II as well (*p* < 0.001). On another hand, when endocarditis was the underlying heart valve disease requiring SAVR, significantly more MPVs were used. Previous AF or anticoagulant therapy were a rare instance in both groups but were more common in recipients for a BPV in aortic position (direct anticoagulants). Incidentally, the type of valve for SAVR was influenced by associated procedures. Simultaneous mitral valve replacement and tricuspid annuloplasty were more common in case of mechanical SAVR (*p* < 0.0001) in relation to a more diffused and advanced heart valve disease, while associated CABGs were more frequent in case of biological SAVR (*p* < 0.0001) with the effect to avoid, in most cases, a combined life-long antiaggregant-anticoagulant therapy. Together with the fact that more recipients of MPVs had already experienced a previous surgery (*p* < 0.0001), discrepancies in associated procedures may have influenced both mean aortic clamping time and duration of ECC which were significantly increased for SAVR with a MPV. Finally, concerning the choice between a biological or a mechanical SAVR, we observed a centre effect which varied with time especially with regards to patients within the intermediate age of 50–60 years-old (Figure 5).

#### In hospital outcomes

Per operative and in-hospital outcome data according to the type of SAVR are summarized in Table 2 and main outcomes regarding type of SAVR in the class of age 50–60 years-old are presented in Table 3. As a result of being significantly younger with less co-morbidity, early mortality was significantly lower in patients receiving a MPV as predicted by preoperative evaluation with the EuroSCORE II risk-scale. However, observed early mortality was below the one that was predicted by the EuroSCORE II for both type of valvular prosthesis and especially for patients between 50 and 60 years-old for which early outcomes were comparable with either type of SAVR in terms of early mortality and major morbidity. Pericardial revisions for tamponade were significantly more frequent after SAVR with a MPV, however excessive bleedings requiring heterologous blood transfusions were more frequent after SAVR with a BPV. Major thromboembolic complications were equally rare after SAVR no matter the type of valvular prosthesis, permanent strokes were however significantly more frequent in elderly patients. Mean postoperative length of stay was not significantly different though the standard deviations were too wide to allow for any conclusion.

**Table 2:** Surgical characteristics and main in-hospital outcomes according to the type of prosthetic valve.

	Mechanical valves *n* = 8,909	Biological valves *n* = 63,466	*p*
Previous Surgery One, No. (%; 95% CI)	1,062 (11.9; 11.2–12.6)	4,260 (6,7; 6.5–6.9)	<0.0001
Two or More, No. (%; 95% CI)	112 (1.2; 1–1.5)	212 (0.33: 0.29–0.28)	<0.0001
Index Surgery Elective, No. (%; 95% CI)	7,978 (89.5; 87.6–91.5)	55,911 (88.1; 87.3–88.8)	0.17
Urgent, No. (%; 95% CI)	673 (7.5; 6.9–8.1)	5,482 (8.6; 8.4–8.8)	0.001
Emergent, No. (%; 95% CI)	187 (2.1; 1.8–2.4)	1,421 (2.2; 2.1–2.3)	0.4
Associated Procedure CABG, No. (%; 95% CI)	1,368 (15.4; 14.5–16.2)	14,253 (22.5; 22.1–22.8)	<0.0001
Mitral, No. (%; 95% CI)	1,355 (15.2; 14.4–16)	3,929 (6.2; 6–6.3)	<0.0001
Tricuspid, No. (%; 95% CI)	407 (4.6; 4.1–5)	1,432 (2.2; 2.1–2.3)	<0.0001
ECC duration, mean. (SD)	99 (54)	91 (47)	<0.0001
Aortic Cross Clamp Time, mean. (SD)	75 (38)	69 (33)	<0.0001
Permanent Stroke, No. (%; 95% CI)	44 (0.5; 0.3–0.6)	380 (0.6; 0.5–0.7)	0.23
Myocardial infarction, No. (%; 95% CI)	40 (0.4; 0.3–0.6)	199 (0.3; 0.2–0.4)	0.04
Excessive bleeding, No. (%; 95% CI)	364 (4; 3.6–4.5)	3,617 (5.7; 5.5–5.9)	<0.0001
Tamponnade, No. (%; 95% CI)	294 (3.3; 2.9–3.7)	1,494 (2.3; 2.2–2.5)	<0.0001
Post operative AF, No. (%; 95% CI)	427 (4.8; 4.3–5.3)	5,245 (8.2; 8–8.5)	<0.0001
Pace-Maker, No. (%; 95% CI)	263 (2.9; 2.6–3.3)	2,284 (3.6; 3.4–3.7	0.0008
Para-valvular leak >2, No. (%; 95% CI)	63 (0.7; 0.5–0.9)	684 (1; 0.9–1.1)	0.007
Postop. length of stay, mean. (SD)	15.8 (8.7)	15.8 (8.6)	1
In-hospital mortality, No. (%; 95% CI)	181 (2; 1.7–2.3)	1,512 (2.4; 2.3–2.5)	0.04
Observed/expected mortality	0.72	0.6	–

**Table 3:** Main in-hospital outcomes according to the type of valve for patients between 50 and 60 years-old.

	Mechanical 3,032	Biological 2,914	Total 5,946	*p*
Permanent Stroke, No. (%)	15 (0.5)	21 (0.7)	36	0.26
Myocardial infarction, No. (%)	12 (0.4)	6 (0.2)	18	0.18
Excessive bleeding, No. (%)	135 (4.4)	211 (7.2)	346	<0.001
Tamponnade, No. (%)	101 (3.3)	83 (2.8)	184	0.28
Post operative AF, No. (%)	161 (5.3)	193 (6.6)	354	0.03
Pace-Maker, No. (%)	79 (2.6)	64 (2.1)	143	0.3
Para-valvular leak >2, No. (%)	26 (0.8)	54 (1.8)	80	0.0009
In-hospital mortality, No. (%)	41 (1.3)	53 (1.8)	94	0.14
Observed/expected mortality	0.68	0.61	0.61	–

## Discussion

Our study reveals a pronounced tendency towards implanting much more BPVs than MPVs during the last 15 years for SAVR in a large French multi centric population of patients. This was expected with regards of our population's age characteristics which match with demography of Western countries. Considering that 75% of patients were over 65-years old, this tendency is in line with both ESC and AHA-ACC recommendations (5, 7). Because, the durability of most BPVs exceeds life expectancy after 65 years-old (1), redo-valve surgery in elderly are generally due to non-structural prosthetic valve dysfunctions or endocarditis (11). However surgeons should be aware that, especially with patients who have more than 10 years of life expectancy, the risk of tissue valve degeneration should be minimized, and that the possibility of a future ViV TAVR should be planed during the first operation (12). At least for such patients, surgeons should therefore choose a BPV for which the long-term durability is well documented and should implant a valve with a diameter at least 23 mm (13), preferably avoiding those prostheses with an inner stent (14). Those general considerations might be even more crucial in patients under 50-years-old, 38% of which received a BPV for SAVR, a percentage stable enough throughout the time of our study.

Most importantly, we found that BPVs recently tended to be preferred over MPVs in patients between 50 and 60 years-old, a trend which is not observed to this extent in some other western countries (15). Added to patients aged from 60 to 65 years-old, they represent the so called middle-aged patients which represent a significant proportion (20%) of the overall population included in our study. In those middle age patients biological SAVR were predominantly implanted. Presumably, beside usual contra-indications to long-term anticoagulant therapy, this reflects an evolution in patient's personal choice. It is certainly also supported by the informations given to the patient by physicians. Those informations are generally based on the notions that, (i) for middle-aged patients, life expectancy post SAVR is not influenced by the type of valvular prosthesis (16), (ii) the quality of life for middle-aged patients is improved with BPV by avoiding life-long anticoagulant therapy and (iii) even though the durability of the biological prosthesis could not be extended despite promising advances, there is an on-going evidence that a degenerated valve could be treated either with a low-risk redo surgery or with a ViV-TAVR.

It is debatable that this given information reflects evidences from the most recent literature. Looking at the hard end-point of late mortality, a recent large hazard ratio meta-analysis including 16,876 patients found a higher late mortality after SAVR with BPVs (hazard ratio: 1.22, 95% CI: 1.00–1.49) for patients younger than 70 years of age (17). Table 4 resumes the most important findings regarding mortality and morbidity outcomes of SAVR with both types of valves in recent comparative large series and totalizing 11,376 middle-aged patients (16, 19–21). Another recent meta-analysis (22) focussing on the risk of prosthetic endocarditis and including 43,941 found that the risk of secondary endocarditis was significantly higher with BPVs than with MPVs. Therefore to ensure that an accurate information has been delivered, the fact that their choice to receive a biological SAVR might result into a reduced life expectancy and a higher risk of endocarditis should not be hidden to middle-aged patients.

**Table 4:** Hazard ratio (*p*-value) of main outcomes after SAVR with MPVs versus BPVs in most recent comparative large studies targeting at middle aged patients.

Study	*n* *	Matching	Follow-up	Death	Stroke	Major bleeding	Redo surgery
Chiang (2014) (16)	2,002	Propensity scoring	15 years	0.97 (0.74)	1.04 (0.84)	1.75 (0.001)	0.52 (0.001)
Glaser (2016) (18)	2,002	Propensity scoring	15 years	0.71 (0.006)	1.04 (0.84)	2.04 (<0.001)	0.42 (0.001)
Goldstone (2017) (19)	3,608	Inverse probability	15 years	0.81 (0.03)	(<0.05)**	(<0.05)**	–
Kytö (2020) (20)	1,152	Propensity scoring	10 years	0.72 (0.03)	1.29 (0.15)	1.19 (0.4)	0.3 (0.009)
Traxler (2022) (21)	2,612	None***	10 years	0.59 (<0.001)	1.04 (0.82)	–	0.3 (0.005)

* Number of middle age patients included in the study, HR refer to this sub-population.

** HR is not indicated but a higher significant risk is mentioned.

*** Multivariable Cox regression.

Besides, considering tissue valve failure, physicians should be aware of a possible subsequent valve re-replacement when index surgery is performed before 60 years-old. This eventuality is easy to conceive since life expectancy then exceeds BPVs' durability (11). More, the procedural risk associated with aortic valve surgical re-replacement remains consistent in state or nation-wide registries (16, 20, 11, 23) and cardiac reoperations in octogenarians have a bad prognosis (24). A nation-wide study from France has shown a lower early mortality with ViV-TAVR as compared to re-SAVR (25). This reduced short-term mortality has been confirmed by a recent meta-analysis (26) but this latter also found no differences between ViV-TAVR and re-SAVR in all-cause and cardiovascular mortality at midterm follow-up. Therefore, as for the final assessment of durability for the newest BPVs in non-elderly patients, we still have to wait to know how the widespread use of ViV-TAVR may positively impact the prospects for biological-SAVR in the middle-age population.

Considering the growing evidence that MPVs might confer a survival advantage in middle-aged patients requiring SAVR, it has to be acknowledged that, despite the observed practice in France, present time is too soon to plea for an extensive use of biological SAVR in patients under 60 years-old. This is in phase with the reappraisal of MPVs use that is observed in the latest evolution of the STS database (27). Despite the ongoing research of an ideal valve that would combine a durability over two-decades and a total freedom from long-term anti-coagulant therapy, and while waiting for late results after the Ozaki technique (28), extending indications of aortic valve repair or Ross procedure towards 50–60 years-old very selected patients might represent a valid option.

## Limitations

Our survey was restricted to a list of solicited centres which actively participate to the EPICARD national database and which volunteered to participate. The survey was closed when the number of 100,000 patients receiving heart valve surgery was obtained. Therefore it might be that a true nation-wide study including all French centres would have shown slightly different results, though it is unlikely that the tendency towards BPVs in middle-aged and young patients requiring a SAVR would have changed much. The EPICARD database is purely declarative and it might be that bad outcomes after surgery were under-reported. Items of the database include data from follow-up, however too much missing data made us unable to report on long term results. Therefore extrapolating from literature is the only option to anticipate what will be the consequences on late survival of middle-aged patients operated-on with the standards of actual practice. Finally the interpretation of the results and the considerations elaborated in the discussion engage the only responsibility of the authors and do not reflect personal opinion of the numerous surgeons who performed SAVRs included in this survey.

## Conclusions

In a large contemporary French patient population, real world practice showed a recent shift towards a lower age-threshold for biological SAVR as compared to what would suggest contemporary guidelines. For those young and middle aged patients who prefer to avoid long term vitamin K antagonists with the hope to return to a normal live, evidences that MPVs in aortic position might confer a survival advantage should be explicitly discussed to give the patient an up-to-date evidence based information as required by contemporary guidelines (5, 6).

## Data Availability

The data analyzed in this study is subject to the following licenses/restrictions: The dataset is the property of the French Society of Thoracic and Cardiovascular Surgery. Requests to access these datasets should be directed to Mme Valérie LEBORGNE, valerie.leborgne@specialitesmedicales.org.

## Author's note

This paper has been presented at the ESC Congress 2023, Amsterdam.

## Member of the EpiCard investigators

Collaborators: EpiCard investigators participating to the Carbon-Free Survey: Paul Achouh, MD, HEGP University-Hospital, Paris, France; Christophe Baufreton, MD, PhD, University Hospital of Angers, Angers, France; Eric Bezon, MD, PhD, Brest University Hospital, Brest, France; Olivier Bouchot, MD, PhD, Dijon University Hospital, Dijon, France; Thierry Bourguignon, MD, PhD, Hôpital Trousseau, Tours, France; Lionel Camilleri, MD, PhD, Clermont-Ferrand Universitary Hospital, Clermont-Ferrand, France; Thierry Caus, MD, PhD, University Hospital of Amiens, Amiens, France; Nicolas Chavanis, MD, Centre Hospitalier Annecy Genevois, Annecy, France; Frédéric Collart, Timone's Hospital, Marseille France; Pierre Corbi, MD, PhD, University Hospital of Poitiers, Poitiers, France; Alain Curtil, MD, Clinique de la Sauvegarde, Lyon, France; Jean Louis De Brux, MD, PhD, Angers University Hospital, Angers, France; Roland Demaria, MD, PhD, Montpellier University Hospital, Montpellier, France; François Bouchard, MD, Rouen University Hospital, Rouen, France; Jean-Marc Frapier, MD, PhD (in memory of), Montpellier University Hospital, Montpellier, France; Jean-Philippe Frieh, MD, Clinique du Tonkin, Villeurbanne, France; Louis Labrousse, MD, PhD, Hôpital du Haut Levêque, Bordeaux, France; Pascal Leprince, MD, PhD, Pitie-Salpétrière Hospital and Sorbonne University, Paris, France; Bertrand Marcheix, MD, PhD, Hôpital de Rangeuil, University Hospital of Toulouse, Toulouse, France; Jean Philippe Mazzucotelli, MD, PhD, Hôpitaux Universitaires de Strasbourg, Strasbourg, Joseph Nader, MD, PhD, Clinique du Millénaire, Montpellier, France; Jean-Christian Roussel, MD, PhD, Nord Laennec University Hospital, Nantes, France; Vito Giovanni Ruggieri, MD, PhD, Reims University Hospital, Reims, France; Michel Tapia, Institut Arnault Tzanck, Saint Laurent du Var, France; Jean-Philippe Verhoye, MD, PhD, Rennes University Hospital, Rennes, France; Andre Vincentelli, MD, PhD, University Hospital of Lille, Lille, France.
